# Neuroserpin Differentiates Between Forms of Tissue Type Plasminogen Activator via pH Dependent Deacylation

**DOI:** 10.3389/fncel.2016.00154

**Published:** 2016-06-15

**Authors:** Karen-Sue B. Carlson, Lan Nguyen, Kat Schwartz, Daniel A. Lawrence, Bradford S. Schwartz

**Affiliations:** ^1^Department of Biomolecular Chemistry, University of Wisconsin, MadisonWI, USA; ^2^Medical Scientist Training Program, University of Wisconsin, MadisonWI, USA; ^3^Departments of Biochemistry and Medicine, University of Illinois, UrbanaIL, USA; ^4^Departments of Medicine and Molecular and Integrative Physiology, University of Michigan, Ann ArborMI, USA

**Keywords:** neuroserpin, tissue plasminogen activator, serpin, serine protease, decacylation

## Abstract

Tissue-type plasminogen activator (t-PA), initially characterized for its critical role in fibrinolysis, also has key functions in both physiologic and pathologic processes in the CNS. Neuroserpin (NSP) is a t-PA specific serine protease inhibitor (serpin) found almost exclusively in the CNS that regulates t-PA’s proteolytic activity and protects against t-PA mediated seizure propagation and blood–brain barrier disruption. This report demonstrates that NSP inhibition of t-PA varies profoundly as a function of pH within the biologically relevant pH range for the CNS, and reflects the stability, rather than the formation of NSP: t-PA acyl-enzyme complexes. Moreover, NSP differentiates between the zymogen-like single chain form (single chain t-PA, sct-PA) and the mature protease form (two chain t-PA, tct-PA) of t-PA, demonstrating different pH profiles for protease inhibition, different pH ranges over which catalytic deacylation occurs, and different pH dependent profiles of deacylation rates for each form of t-PA. NSP’s pH dependent inhibition of t-PA is not accounted for by differential acylation, and is specific for the NSP-t-PA serpin-protease pair. These results demonstrate a novel mechanism for the differential regulation of the two forms of t-PA in the CNS, and suggest a potential specific regulatory role for CNS pH in controlling t-PA proteolytic activity.

## Introduction

Tissue t-PA was initially described as an intravascular protease capable of initiating fibrinolysis by activation of plasminogen to plasmin (Collen, 1980). However, t-PA is also an important extravascular protease in the central nervous system (CNS). T-PA participates in axonal remodeling, neuronal plasticity, long-term potentiation (Seeds et al., 1995), propagation of excitotoxin-induced seizures (Qian et al., 1993; Tsirka et al., 1995; Endo et al., 1999; Wu et al., 2000; Yepes et al., 2002; Pawlak et al., 2005; Fredriksson et al., 2015), progression of cerebral infarct volume during ischemic stroke (Wang et al., 1998; Kano et al., 2000; Yepes et al., 2000), and regulation of blood brain barrier permeability in a number of pathologic conditions (Yepes et al., 2003; Su et al., 2008; Fredriksson et al., 2015; Lewandowski et al., 2016). The molecular mechanisms for these physiologic processes include both proteolytic and non-enzymatic t-PA properties.

Similar to other serine proteases, t-PA is synthesized and secreted as a zymogen-like molecule, sct-PA. However, in contrast to other serine protease zymogens, sct-PA exhibits appreciable proteolytic activity, by some measures demonstrating 25% of the activity of the fully mature protease, two chain t-PA (tct-PA) (Boose et al., 1989; Tachias and Madison, 1997). The primary substrates of t-PA in the CNS are plasminogen and PDGF-CC (Su et al., 2008; Fredriksson et al., 2015). Studies have also suggested that the GluN1 subunit of the NMDA receptor can be cleaved by t-PA. However, the direct cleavage of the NMDA receptor by t-PA is controversial (Matys and Strickland, 2003; Vivien et al., 2003; Samson et al., 2008; Yuan et al., 2009). It has also been proposed that NMDA receptor mediated excitotoxicity is triggered by sct-PA, and not tct-PA (Parcq et al., 2012; Bertrand et al., 2015), identifying a potential proteolytic regulatory mechanism based on the state of the t-PA molecule.

The control of t-PA enzymatic activity is important for regulation of tPA protease-dependent processes. There are two primary inhibitors of t-PA in the CNS, PAI-1 (Colucci et al., 1986) and NSP (Osterwalder et al., 1996; Hastings et al., 1997; Krueger et al., 1997; Osterwalder et al., 1998; Fredriksson et al., 2015). Although the amount of PAI-1 in the CNS is small (Yamamoto et al., 1994; Fredriksson et al., 2015), the molecular chemistry by which it inhibits t-PA enzymatic activity is well characterized and is consistent with the current understanding of serine protease inhibition by a cognate serpin.

Plasminogen activator inhibitor type-1 functions via the established serpin mechanism, which involves presentation of a specific peptide bond within the serpin reactive center loop as a pseudosubstrate for the protease. The protease initiates cleavage as it would with a substrate, forming an acyl-enzyme intermediate with the P_1_ residue of the serpin (Lawrence et al., 1995; Olson et al., 1995; Wilczynska et al., 1995). However, before the acyl-enzyme can undergo deacylation to complete cleavage of the scissile bond, the serpin undergoes structural re-arrangement, translocating the protease, still covalently linked to the P_1_ residue, 70 Å to the opposite pole of the serpin (Stratikos and Gettins, 1997; Stratikos and Gettins, 1999; Huntington et al., 2000; Dementiev et al., 2006). Within the acyl-enzyme complex, the structure of the protease active site geometry is distorted such that catalytic deacylation can no longer take occur (Huntington et al., 2000; Dementiev et al., 2006). The rearranged acyl-enzyme complex exhibits novel molecular determinants that subsequently mediate specific cell-surface receptor mediated internalization and degradation of the protease-serpin complex (Horn et al., 1998; Stefansson et al., 1998). Hence, protease inhibition by a serpin results in clearance and degradation of both molecules, constituting effectively irreversible inhibition.

Our earlier studies noted a potential mechanistic difference between NSP and PAI-1 inhibition of tPA (Barker-Carlson et al., 2002). *In vitro* biochemical studies demonstrated only transient inhibition of t-PA by NSP due to increased efficiency of catalytic NSP-t-PA deacylation compared to PAI-1-t-PA acyl-enzyme complexes (Barker-Carlson et al., 2002). The shorter period of stable t-PA inhibition by NSP appears to be biologically meaningful however, as NSP interaction with t-PA *in vivo* has been identified as a significant negative-regulator of t-PA mediated effects on ischemic stroke (Yepes et al., 2000), seizure propagation (Yepes et al., 2002) and seizure induced blood brain barrier dysregulation (Fredriksson et al., 2015). Interestingly, in both the above noted pathologic states, ischemic stroke and seizure, the pH of the cerebral spinal fluid decreases to levels that affect the function of human serine proteases (Siesjo, 1985; von Hanwehr et al., 1986).

To determine whether the observed differences in NSP inhibition of t-PA between the *in vitro* and *in vivo* systems might reflect an effect of pH (regulated at 7.2–7.4 *in vitro*, and decreased to <6.8 *in vivo*) we investigated whether the pH in which NSP-tPA interactions were studied might be a differentiating factor in these assay systems. The data presented in this paper demonstrate that although NSP mediated inhibition of t-PA is less stable than PAI-1 inhibition of t-PA at physiologically neutral pH, NSP mediated inhibition is modulated by biologically relevant changes in pH, which result in changes in the rate of catalytic deacylation of NSP-t-PA complexes. Moreover, pH differences also allow NSP to differentiate between sct-PA and tct-PA. This leads us to hypothesize that variations in pH may play a heretofore unrecognized regulatory role in CNS processes that involve t-PA.

## Materials and Methods

### Proteins and Reagents

Tissue-t-PA was purchased from Calbiochem (Leola, CA, USA) and Biopool (Sweden) (>95%, and >99% sct-PA, respectively, as assessed by SDS-PAGE under reducing conditions followed by silver staining); tct-PA was generated by treatment of sct-PA with plasmin-linked sepharose for a time determined to yield complete conversion of sct-PA to tct-PA as assessed by SDS-PAGE as above (Schwartz and Espana, 1999). Plasmin, spectrozyme t-PA, and traysolol were purchased from American Diagnostica (Greenwich, CT, USA), and two-chain urokinase (tcu-PA) was obtained from Dr. Gene Murano (Monsanto, St. Louis, USA). Cell culture grade bovine serum albumin (BSA) was purchased from Sigma (St. Louis, MO, USA). Recombinant human PAI-1 (14-1b stabilized mutant) was produced in a bacterial expression system as previously described (Berkenpas et al., 1995). Polyclonal rabbit antibody against PAI-1 was a generous gift of Dr. Peter Andreasen (Aarhus University, Denmark) (Zeheb et al., 1987). Polyclonal antibody against NSP was generated in rabbits (Hastings et al., 1997) and HRP-conjugated goat antibody against rabbit IgG was purchased from Pierce (Rockford, IL, USA) and Jackson ImmunoResearch Laboratories (West Grove, PA, USA). Human NSP cDNA was obtained from Human Genome Sciences (Rockville, MD, USA) and NSP was expressed in a bacculovirus system (Hastings et al., 1997).

### Electrophoresis and Western Blot Analysis

Both the mini-protean three apparatus used for electrophoresis and protein transfer, as well as electrophoresis reagents were from BioRad (Hercules, CA, USA). Polyacrylamide gels were cast with 3% stacking and 10% separating gels and Western blotting for NSP or PAI-1 was carried out as previously described (Barker-Carlson et al., 2002; Li et al., 2008). Analysis of immunoblots using known amounts of NSP that was intact, cleaved, or in complex with sct-PA demonstrated no detectable difference in epitope detection among the states of NSP (Barker-Carlson et al., 2002). As noted in our previous paper (Barker-Carlson et al., 2002) these reagents were validating as demonstrating no detectable difference in epitope detection among the states of NSP. This information is included in the “Methods” section. A standard curve for NSP-sc-tPA complex detection using this methodology is also included in **Supplemental Figures S1A,B**. Kodak 1D Software was used to image all films, and Prism 3.0 or 4.0 software were used for data analysis as indicated.

### Assessment of Serpin-PA Complex Stability as a Function of pH

Seventy nanometer NSP, or 140 nM PAI-1 was incubated at 37°C for 5 and 15 min, respectively, with equimolar amounts of the indicated forms of t-PA, or tcu-PA in 0.1 M NaCl, 0.001 M sodium phosphate, pH 7.2, and 100 μg/mL BSA. Reactions with NSP included 0.1% Triton X-100, and those with PAI-1 included 1,000 U/mL trasylol. Preliminary experiments demonstrated that Triton X-100 had no influence on the function of NSP. Reactions were then diluted fivefold into a series of 0.1 M sodium phosphate buffers at the indicated pH values, from 6.0 to 8.0, and incubated for 30 min (NSP) or 90 min (PAI-1) at 37°C. Reactions were quenched by addition of SDS-sample buffer, boiled and subjected to SDS-PAGE under reducing conditions, followed by Western blot analysis for NSP or PAI-1. The resulting immunoblot images were analyzed to quantify remaining acyl-enzyme serpin-protease complexes as a fraction of the complexes at time zero.

### Effect of pH on Serpin Inhibition of t-PA Activity

One hundred and thirty-seven nanometer sct-PA or tct-PA was pre-incubated with 302 nM NSP or 200 nM PAI-1 for 5 (sct-PA) or 2 min (tct-PA), at 37°C in 0.15 M NaCl, 0.001 M Tris, pH 7.2 with 100 μg/mL BSA. Reactions were then diluted fivefold into buffers containing 0.05 M NaCl, 0.1 M Tris, 100 μg/mL BSA at the pH’s indicated, and incubated a further 50 min (sct-PA) or (tct-PA), or 30 min at 37°C. Reactions with NSP contained 0.1% Triton X-100. Residual t-PA activity was determined by adding spectrozyme t-PA at a final concentration of 1.5 mM using the kinetic analysis module in a Beckman DU-640 spectrophotometer. Inhibition of t-PA enzymatic activity was calculated as shown in Equation 1.

Equation 1:

$$100-100(t-PA-{serpin}_{pHx}/t-{PA}_{pHx})=\%t-PA\,\;inhibition$$

Where t-PA-serpin_pHx_ represents the rate of chromogenic substrate cleavage in reactions containing t-PA and the indicated serpin at a specific pH between 6.0 and 8.0, and t-PA_pHx_ represents the rate of chromogenic substrate cleavage by t-PA at the same pH in the absence of serpin.

Neuroserpin-t-PA (either form of t-PA) acyl-enzyme complexes demonstrated no catalytic deacylation at pH 6.0, thus inhibition of t-PA activity at pH 6.0 was designated 100%, and inhibition at other pH’s was expressed relative to that at pH 6.0, as shown in Equation 2.

Equation 2:

$$\%t-{PA\,\;inhibition}_{pHx}/\%{t-PA\,\;inhibition}_{pH6.0}=t-PA\,\;inhibition\,\;relative\,\;to\,\;that\,\;at\,\;pH\,\;6.0$$

Where % t-PA inhibition pH_x_ represents the percent inhibition of t-PA at the indicated pH, and % t-PA inhibition _pH6.0_ is the percent inhibition at pH 6.0, both determined as in Equation 1. The pH profile for the relative inhibition of t-PA by NSP was generated and fit to a sigmoidal curve with variable slope non-linear regression using Prism 4.0 software.

### Effect of pH on the Rate of NSP-t-PA Acyl-Enzyme Complex Deacylation

Formation of acyl-enzyme complexes between each form of t-PA and NSP progresses more rapidly than does deacylation (Barker-Carlson et al., 2002). Preliminary experiments defined conditions wherein NSP-t-PA acyl-enzyme complex formation had gone to completion, and therefore the decrement in NSP within those complexes over time could be quantified. Equimolar concentrations (70 nM) of NSP and sct-PA, or tct-PA were incubated for 10 or 2 min, respectively, at 37°C in 0.1 M NaCl, 0.001 M sodium phosphate, pH 7.2, with 100 μg/mL BSA and 0.1% Triton X-100. Reactions were then diluted fivefold (final concentration of t-PA and NSP, 14 mM) into 0.1 M sodium phosphate buffer, at the indicated pH between 6.0 and 7.6, containing 100 μg/mL BSA and 0.1% Triton X-100, and incubated at 37°C. At sequential times, aliquots were removed and quenched by addition of SDS-sample buffer, boiled, and analyzed by SDS-PAGE under reducing conditions, followed by Western blot analysis of NSP antigen. The deacylation rate constant at each pH was determined by fitting the data for the decay of NSP-t-PA acyl-enzyme complexes, which was linear with respect to time at each pH tested, to the equation for unimolecular decay (Equation 3).

Equation 3:

$${In\left[NSP-t-PA\right]}_{t}={In\left[NSP-t-PA\right]}_{o}-k_{3}t$$

Where [NSP-t-PA]_t_ is the concentration of NSP-t-PA acyl-enzyme complexes at time t, [NSP-t-PA]_o_ is the initial concentration of complexes, and k_3_ is the deacylation rate constant (Schechter et al., 1997; Plotnick et al., 2002a,b; Schechter and Plotnick, 2004).

## Results

Previous work from our labs revealed that the interaction between t-PA and NSP did not form the prototypically stable serpin-protease acyl-enzyme complexes as seen with t-PA and its other cognate serpin, PAI-1. Rather, formation of acyl-enzyme complexes between t-PA and NSP occurred readily, but was followed by rapid deacylation yielding cleaved serpin and active enzyme (Barker-Carlson et al., 2002). In contrast to these unstable *in vitro* NSP-t-PA complexes, administration of NSP to animals undergoing experimental stroke or seizure yielded evidence of t-PA inhibition (Osterwalder et al., 1998; Yepes et al., 2000, 2002). Unlike *in vitro* biochemical experiments which are generally performed at pH 7.2–7.4, the CNS is relatively poorly buffered and its pH drops to as low as 6.0 during pathologies wherein t-PA plays a role (Meldrum and Brierley, 1973; Meldrum and Horton, 1973; Aminoff and Simon, 1980; Siesjo, 1985, 1986, 1992; von Hanwehr et al., 1986; Bereczki and Csiba, 1993; Meric et al., 1994). We hypothesized that the rapid drop in CNS pH documented to occur following the onset of stroke or seizure might influence the stability of NSP-t-PA acyl-enzyme complexes, and the accompanying inhibition of t-PA enzymatic activity.

**FIGURE 1:**
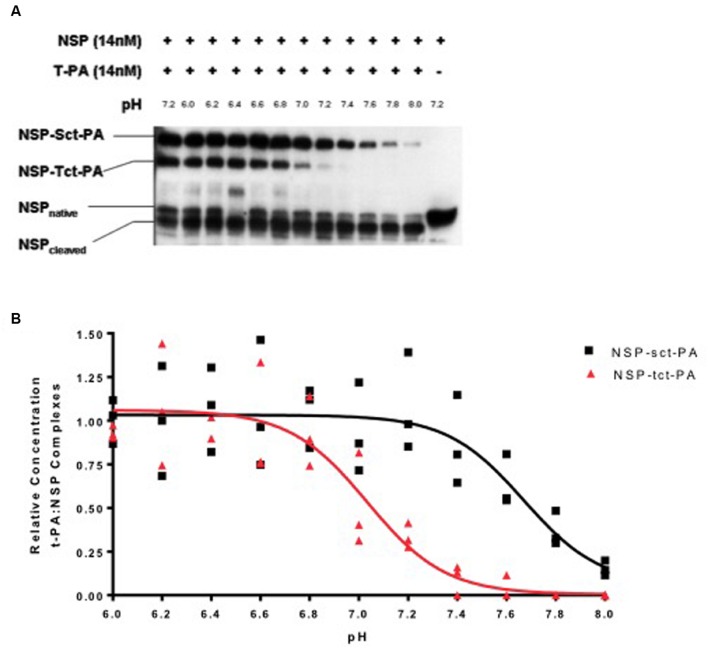
**Stability of t-PA-NSP acyl-enzyme complexes is pH dependent.** **(A)** Following initial formation of NSP t-PA complexes with NSP at pH 7.2, reactions were diluted into buffers between 6.0 and 8.0 and incubated at 37°C for an additional 30 min as detailed in Methods, then analyzed by SDS-PAGE and Western blotting for NSP. The far left lane illustrates the quantity of NSP-t-PA complexes present at the beginning of the 30-min incubation (maximal complex). The far right lane contains NSP that was not reacted with t-PA. The remaining lanes show the reaction products at the end of the 30-min incubation at each noted pH. The identities of the individual bands are indicated on the left side of the gel. For ease of comparison, reactions with sct-PA and tct-PA were run in the same wells, since the NSP-t-PA acyl-enzyme complexes involving each form of t-PA are easily resolved. Identical results were obtained when reactions between NSP and each form of NSP were analyzed separately. **(B)** The ratio of t-PA-NSP complex remaining at the end of the 30-min incubation at the indicated pH, to the amount of complex present immediately before dilution into various pH buffers was graphed as a function of the pH into which the reactions were diluted. Triangles: NSP-tct-PA complexes; Squares: NSP-sct-PA complexes. Data are presented as points that represent the individual values, from 3 independent experiments, and lines that represent the means of those values.

To test this hypothesis, NSP was allowed to complex with either sct-PA or tct-PA at pH 7.2, aliquots were then brought to different pH’s across the range of 6.0–8.0 and incubated a further 30 min at 37^o^C. The reactions were then analyzed by SDS-PAGE and Western blotting for NSP antigen, and the amount of NSP that remained in complex was compared to the amount initially present in acyl-enzyme complex. **Figure 1** demonstrates several findings. First, the effect of pH on acyl-enzyme stability is clearly different for NSP in complex with sct-PA compared to NSP in complex with tct-PA. Second, the difference in acyl-enzyme stability between NSP in complex with the two forms of t-PA is greatest at physiologic pH, 7.4. Third, although acyl-enzyme complexes between NSP and tct-PA are unstable at pH 7.4 suggesting tct-PA is free to exert proteolytic activity at physiologic pH, slight acidic shifts result in significant stabilization of these complexes.

The loss of acyl-enzyme complexes does indeed signify catalytic deacylation, as the appearance of cleaved NSP accounts for the reduction in acyl-enzyme-complex with t-PA (**Figure 1A**), and the pH range in which this occurs is not compatible with hydroxide mediated deacylation (Calugaru et al., 2001). These findings are consistent with the mechanism for catalytic deacylation of an acyl-enzyme intermediate of a serine proteinase, which requires that the active site histidine within the catalytic triad accept a proton (Plotnick et al., 2002a). At lower pH values, the histidine is likely already protonated, and thus catalytic deacylation cannot proceed. These data are also consistent with previous observations that at pH 7.2, deacylation readily occurs (Barker-Carlson et al., 2002).

**FIGURE 2:**
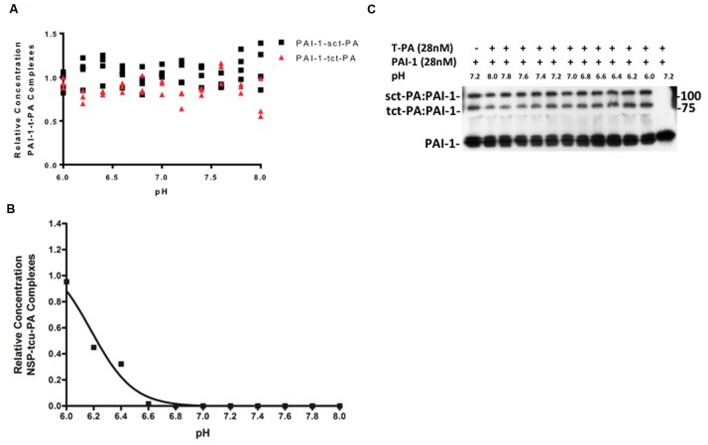
**(A,B)** pH dependent stability of acyl-enzyme complexes is specific for NSP-t-PA complexes. PAI-1-t-PA complex stability was assayed in a manner analogous to NSP-t-PA complex stability, as described in Methods. The ratio of PAI-1-t-PA complex remaining at the end of the 90-min incubation to the amount of complex present immediately before dilution into various pH buffers, was graphed as a function of the pH into which the reactions were diluted. Triangles: PAI-1-tct-PA complexes; Squares: PAI-1-sct-PA complexes. Data are presented as points that represent the individual values, from 3 independent experiments Note that incubation of PAI-1-t-PA complexes at various pH’s was for 90 min, compared to 30 min for NSP-t-PA complexes, further illustrating the difference in stability between the acyl-enzyme complexes. Panel **(B)** is a representative western blot from which the data were derived and quantified for **(A)**. **(C)** tcu-PA and NSP were incubated in buffer pH 7.2 for 1 min at 37°C, diluted fivefold into buffers at the indicated pH, and incubated at 37°C for 3 h. Samples were then analyzed by SDS-PAGE and western blotting for NSP as described in Methods. The ratio of the concentration of the tcu-PA-NSP complexes remaining after the 3-h incubation at each pH to the concentration of complex present immediately before dilution into various pH buffers was graphed against the pH into which the reactions were diluted.

These effects do not seem to be general characteristics of t-PA-serpin interactions, as PAI-1-t-PA acyl-enzyme complexes are stable across the pH range in which NSP-t-PA complexes undergo catalytic deacylation, and no differential stability between PAI-1-sct-PA complexes and PAI-1-tct-PA complexes was evident (**Figures 2A,B**). In addition, this pattern of acyl-enzyme instability does not characterize NSP interactions with other proteases closely related to t-PA. Under no circumstances tested were acyl-enzyme complexes between plasmin and NSP detected, but instead NSP acted as a pure plasmin substrate across the pH range tested (data not shown). Two-chain u-PA cleaved NSP with no detectable acyl-enzyme intermediate at physiologic pH, essentially treating the serpin as a substrate. However, rapid adjustment of conditions to very acidic pH allowed detection of stable tcu-PA-NSP acyl-enzyme complexes (**Figure 2C**). This is consistent with the pattern observed for non-cognate protease-serpin pairs (Schechter et al., 1997; Plotnick et al., 2002a,b; Schechter and Plotnick, 2004). Although single chain u-PA forms acyl-enzyme complexes with PAI-1 (Manchanda and Schwartz, 1995), no complexes between single chain u-PA and NSP were detected (data not shown). Hence, the regulation of NSP inhibition of t-PA over the narrow pH range known to occur in physiologic and pathologic processes in the mammalian CNS seems selective for this protease-serpin pair. This is in marked contrast to t-PA and u-PA reacting with the serpin PAI-1, and with the substrate plasminogen (Colucci et al., 1986), and is consistent with the findings of Fredriksson et. al., which suggests that NSP regulates a t-PA mediated process (Fredriksson et al., 2015). The intriguing possibility raised by the present data is that there may be selectivity for regulating the different forms of t-PA under certain biological conditions.

**FIGURE 3:**
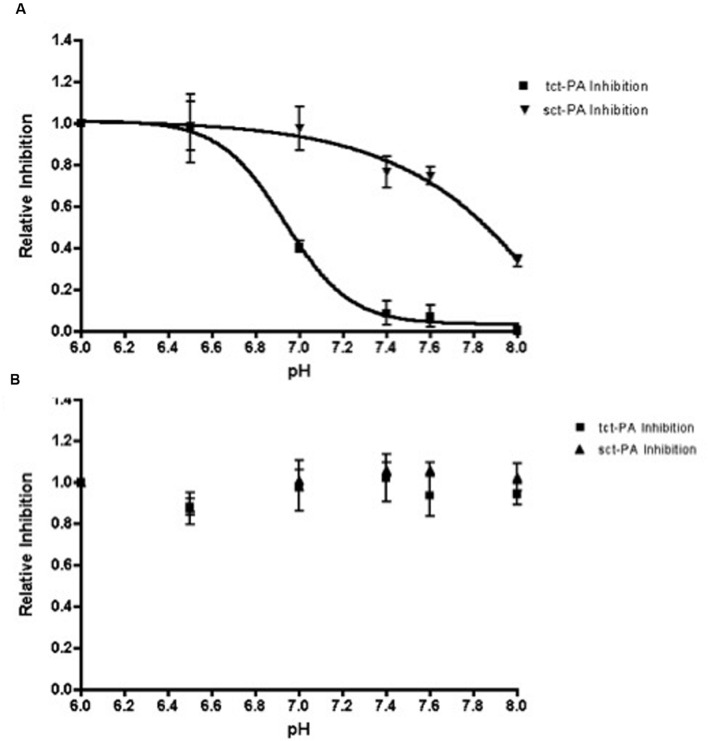
**Inhibition of t-PA enzymatic activity by NSP, but not PAI-1, is dependent on pH.** Sct-PA or tct-PA were pre-incubated with either **(A)** NSP, or **(B)** PAI-1 as detailed in Methods. Reactions were then diluted into buffers with the indicated pH’s as in Methods, and the residual t-PA activity determined by quantifying cleavage of a chromogenic substrate. The per cent t-PA inhibition at a given pH was compared to the maximum inhibition (observed at pH 6.0) to yield the relative inhibition of each form of t-PA, which is graphed as a function of pH. Triangles: Serpin-sct-PA complexes; Squares: Serpin-tct-PA complexes. Data represent the mean and SEM from 3 independent experiments.

Stability of an acyl-enzyme complex suggests the active site architecture of the protease remains distorted within the complex, preventing catalytic deacylation (Lawrence et al., 1990; Huntington et al., 2000; Dementiev et al., 2006). Therefore, the pH dependent change in NSP-t-PA acyl-enzyme complex stability should be paralleled by a pH dependent change in t-PA inhibition. To test this, NSP and both forms of t-PA (sct-PA or tct-PA) were incubated at pH 7.2 to allow acyl-enzyme complexes to form. The complexes were then diluted into buffers with pH’s between 6.0 and 8.0, incubated further, and then added to a t-PA sensitive chromogenic substrate to determine residual t-PA catalytic activity, and thus the degree of t-PA inhibition. As seen in **Figure 3**, t-PA inhibition as a function of pH closely paralleled the persistence of NSP-t-PA acyl-enzyme complexes as a function of pH (compare **Figures 1B** and **3A**). In addition, the surprising difference in pH effect on acyl-enzyme complex stability between sct-PA and tct-PA was also seen in pH dependent inhibition of the two forms of t-PA (**Figure 3A**). As was seen with acyl-enzyme complex persistence, this pattern of inhibition was unique to the interaction between t-PA and NSP, as PAI-1 showed no difference in t-PA inhibitory efficiency across the pH range tested, nor did PAI-1 differentiate between the two forms of the enzyme (**Figure 3B**). These data also suggest that the pH dependent difference in acyl-enzyme complex persistence was not a function of an SDS-PAGE based assay system, as the experiments determining inhibition of enzymatic activity contained no denaturants.

**FIGURE 4:**
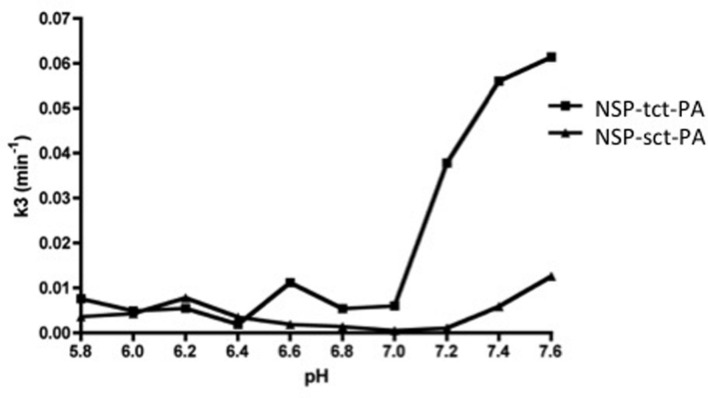
**Differential effects of pH on the deacylation rates of NSP-sct-PA and NSP-tct-PA acyl-enzyme complexes.** Acyl-enzyme complex formation between sct-PA or tct-PA and NSP was allowed to progress to completion at pH 7.2, as determined in preliminary experiments. Individual reactions were then transferred to the indicated pH and the decay of acyl-enzyme complexes followed over time by subjecting aliquots to SDS-PAGE and western blotting for NSP antigen as detailed in Methods. The deacylation rate constant (k_3_) was determined for each pH as described in Methods. Triangles, NSP-sct-PA complexes; Squares, NSP-tct-PA complexes. Data are representative of two independent sets of experiments.

The above data suggest that NSP may have the capacity to differentially inhibit sct-PA and tct-PA via distinct rates of deacylation of the acyl-enzyme complexes once they are formed. To test this hypothesis, NSP-sct-PA and NSP-tct-PA acyl-enzyme complexes were pre-formed, transferred to the indicated pH, and rates of acyl-enzyme complex deacylation determined. To ensure the data were not confounded by the formation of new NSP-t-PA acyl-enzyme complexes, measurement of NSP-t-PA complex decay was only performed at times after all NSP had been incorporated into acyl-enzyme complexes, as assessed by SDS-PAGE and western blotting for NSP. Hence, the reduction in NSP-t-PA complex intensity was an accurate measure of deacylation. As seen in **Figure 4**, the plots of the deacylation rate constants as a function of pH differed between NSP in complex with sct-PA, and tct-PA. In addition, the rate constants were consistent with the pH dependent acyl-enzyme persistence and enzyme inhibition with each form of t-PA (**Figures 1** and **3**). NSP-tct-PA deacylation rates remained low below pH 7.0, the range where acyl-enzyme complexes persisted and tct-PA enzymatic activity was inhibited. As the pH increased above 7.0, the deacylation rate increased (**Figure 4**), with parallel decreases in acyl-enzyme complex persistence (**Figure 1B**) and enzymatic inhibition (**Figure 3A**). For NSP in complex with sct-PA, the increase in deacylation rate occurred at a significantly higher pH, 7.4, again with corresponding changes in acyl-enzyme complex persistence and enzyme inhibition (**Figures 1B** and **3A,** respectively).

## Discussion

Given the relatively steep slope of the pH effect on NSP inhibition of t-PA, and the similar slopes of pH dependent stability for acyl-enzyme complexes containing either form of t-PA (**Figure 1B**), it seems that this pH effect is due to a limited number of residues. The pH range across which NSP-t-PA catalytic deacylation is regulated is consistent with titration of the catalytic triad histidine (HIS 57, chymotrypsin numbering), and suggests significantly different molecular environments for HIS 57 of sct-PA versus tct-PA in complex with NSP, with a different H^+^ ion concentration required to allow initiation of catalytic deacylation. However, once that threshold has been crossed, the similar titration curves for deacylation of NSP in complex with sct-PA or tct-PA suggests a similar process for each form of t-PA.

It is also possible that the distinct pH profiles of deacylation signify differences in a non-active site residue on either molecule important for maintaining t-PA in the deformed conformation (i.e., at a contact point between protease and serpin). For instance, it is possible that as a residue at the interface between t-PA and NSP is deprotonated, the ability of NSP to maintain misalignment of t-PA’s active site architecture is lost, resulting in recovery of the capacity for catalytic deacylation of the acyl-enzyme complex. If so, the pH sensitivity of this residue in NSP-sct-PA complexes must differ from that in NSP-tct-PA complexes. Such a scenario would also yield a difference primarily in the threshold at which deacylation is initiated, with subsequent events being similar between forms of t-PA. It will be important to test these and other hypotheses of the molecular mechanism for the differential pH effects on NSP-t-PA deacylation in future experiments.

This model is consistent with the findings of Calugaru et al. (2001), who observed that in certain non-cognate serpin-protease pairs, Ca^++^ served as an allosteric ligand which restored partial proteolytic capability to the serpin-complexed protease active site, allowing for catalytic deacylation of the serpin-protease intermediate. In the case of NSP-t-PA complexes, there does not appear to be a requirement for an allosteric ligand to bring about a pH responsive conformation; such a conformation appears to have evolved specifically in NSP-t-PA complexes.

An intriguing correlation may be made with recent findings by Lee et al. (2015), who showed that NSP-t-PA complex stability is dependent on evolutionarily conserved residues in NSP, and their findings will inform design of future NSP-mutants to identify the amino acid residues that are ultimately responsible for the pH dependency of NSP-t-PA complex deacylation.

The findings in this report are also consistent with a population of NSP-complexed t-PA molecules that retain some degree of catalytic function in equilibrium with NSP- complexed t-PA molecules exhibiting little or no catalytic function, as hypothesized for unstable serpin-enzyme pairs (Calugaru et al., 2001; Plotnick et al., 2002b). As the somewhat functional t-PA molecules complete the catalytic cleavage of NSP, an essentially irreversible event, the equilibrium model suggests that some molecules in the non-functional conformation then shift to the somewhat functional state, and further catalytic deacylation of complexed NSP occurs. This is depicted in Scheme 1.

Scheme 1:

$$t-{PA}_{D}-NSP\begin{array}{c} \to \\ \leftarrow \end{array}t-{PA}_{F}-NSP\to t-PA+{NSP}_{cleaved}$$

where t-PA_D_ is deformed and catalytically inactive t-PA in complex with NSP, t-PA_F_ is t-PA that has retained some catalytic function in complex with NSP, and NSP_cleaved_ is NSP that has had the cleavage of P_1_–P_1_′ completed. Therefore, the catalytic activity of the t-PA_F_ form of the protease might be pH sensitive (presumably due to titration of HIS 57), or the stabilization of the t-PA_D_-NSP complex might be pH dependent (presumably via optimization of intermolecular contacts).

It is interesting that despite the metabolic fragility of the CNS, the CSF has relatively poor buffering capacity. This suggests that allowing changes in CNS pH may have been preserved through evolution, a concept that is consistent with pH having an important regulatory role in the in this anatomic compartment. The data in this paper suggest that specific regulation of t-PA activity by NSP may be an example of a process regulated by shifts in CNS pH. For instance, neuronal depolarization is accompanied by a flux of hydrogen ions at the synapse, and similarly, secretion of NSP-containing dense core secretory vesicles, which have a pH of 5.0–6.0, would also be expected to transiently lower the local synaptic pH (Loh et al., 1984; Chuang et al., 1999; Parmar et al., 2002; Ishigami et al., 2007). Additional mechanisms of synaptic pH modulation include the Ca+2/H+-ATPase and carbonic anhydrases that are all functional within the microdomain of the synapse (Sinning and Hubner, 2013). A shift in pH within a single synapse could be sufficient to modulate NSP inhibitory stability, resultant t-PA activity, and t-PA-dependent neuronal function for that specific synapse (Qian et al., 1993; Seeds et al., 1995; Frey et al., 1996; Calabresi et al., 2000; Zhuo et al., 2000). These same pH alterations can also be hypothesized to modify NMDA receptor activity (Tang et al., 1990; Traynelis and Cull-Candy, 1990; Sinning and Hubner, 2013), with relatively little effect on AMPA- and kainite receptor function (Lei et al., 2001). Although beyond the scope of this manuscript, it is tempting to speculate that the inhibition of NMDA receptor activity noted with decreased extracellular pH may in part be related to persistence of NSP-t-PA acyl-enzyme complexes, thus altering t-PA interaction with the NR2B subunit of the NMDA receptor (Norris and Strickland, 2007; Parcq et al., 2012).

It is also intriguing to speculate that pH dependent regulation of t-PA proteolytic activity may be operative in pathologic instances such as seizures, where depolarization goes unchecked. The spread of seizures in mice is t-PA dependent (Yepes et al., 2002), and in a murine model of neonatal febrile seizures, hyperventilation driven alkalinization of the CSF was shown to be the trigger for seizures (Schuchmann et al., 2006). This is consistent with the use of hyperventilation to induce seizures during video EEG monitoring to determine seizure focus in the CNS (Guaranha et al., 2005). Perhaps hyperventilation-induced CNS alkalinization results in loss of NSP inhibitory activity, releasing unopposed t-PA activity, thus facilitating seizure spread.

NSP polymerization has also been shown to be dependent on pH (Belorgey et al., 2010, 2011), and NSP is more resistant to polymerization at low pH than is PAI-1 (Ishigami et al., 2007; Takehara et al., 2009; Belorgey et al., 2010). Because available NSP or PAI-1 is a function of the balance between polymerized and free serpin, with only free serpin being able to complex with protease, it is possible that the pH effect described in the present report reflects differences in NSP polymerization, and thus differences in the serpin’s availability to inhibit t-PA. However, deacylation was determined starting with NSP that was already in complex with each form of t-PA (**Figure 4**), something that requires non-polymerized NSP. Hence, the regulatory step described here appears to be separate from the pH effect on NSP polymerization.

It is also worth considering whether differential pH-dependent regulation of sct-PA and tct-PA reflects different functions for each form of the protease in the CNS. This differentiation may reflect one reason t-PA evolved uniquely as a protease with such a remarkably active zymogen, and is consistent with the distinct role of sct-PA in activating NMDA receptor-dependent neurotoxicity (Parcq et al., 2012; Bertrand et al., 2015) Moreover, Parmar et al.’s finding that tct-PA forms acyl-enzyme complexes with NSP at lower pH than does sct-PA (Parmar et al., 2002) is consistent with different molecular environments in the active sites of the two forms of t-PA during acyl-enzyme formation. Those data, combined with the findings in this paper regarding acyl-enzyme stability, strengthen the hypothesis that changes in H+ ion concentrations are important in regulating the interaction of NSP and t-PA.

Importantly, the properties of NSP that make it uniquely suited as a differential regulator for two forms of t-PA in the pH-sensitive environment of the CNS support the conclusion of Fredriksson et al. (2015) that t-PA is a physiologic target of NSP in the CNS. This is further supported by recent findings that there is very little PAI-1 in neuronal tissue of the normal brain, further supporting the important regulatory function of NSP for t-PA inhibition in the extravascular compartment of the CNS (Yamamoto et al., 1994; Fredriksson et al., 2015).

Further studies will be required to define the molecular mechanisms and physiologic import of this novel form of protease regulation.

## Author Contributions

KS-C and LN contributed equally to this manuscript. KS-C, LN, KS, DL, and BS designed and implemented the experiments, and co-wrote this manuscript.

## Conflict of Interest Statement

The authors declare that the research was conducted in the absence of any commercial or financial relationships that could be construed as a potential conflict of interest.

## Abbreviations

NSP: neuroserpin
PAI-1: plasminogen activator inhibitor type-1
sct-PA: single chain t-PA
t-PA: tissue-plasminogen activator
tct-PA: two-chain t-PA
u-PA: urokinase plasminogen activator
