# Supplementary material for: Neuroserpin Differentiates Between Forms of Tissue Type Plasminogen Activator via pH Dependent Deacylation
Source: Front Cell Neurosci. 2016 Jun 15;10:154. doi: 10.3389/fncel.2016.00154 (PMC4908126; doi:10.3389/fncel.2016.00154)
Supplement: Supplementary file 1 [file Data_Sheet_1.PDF]

Supplemental Figure 1

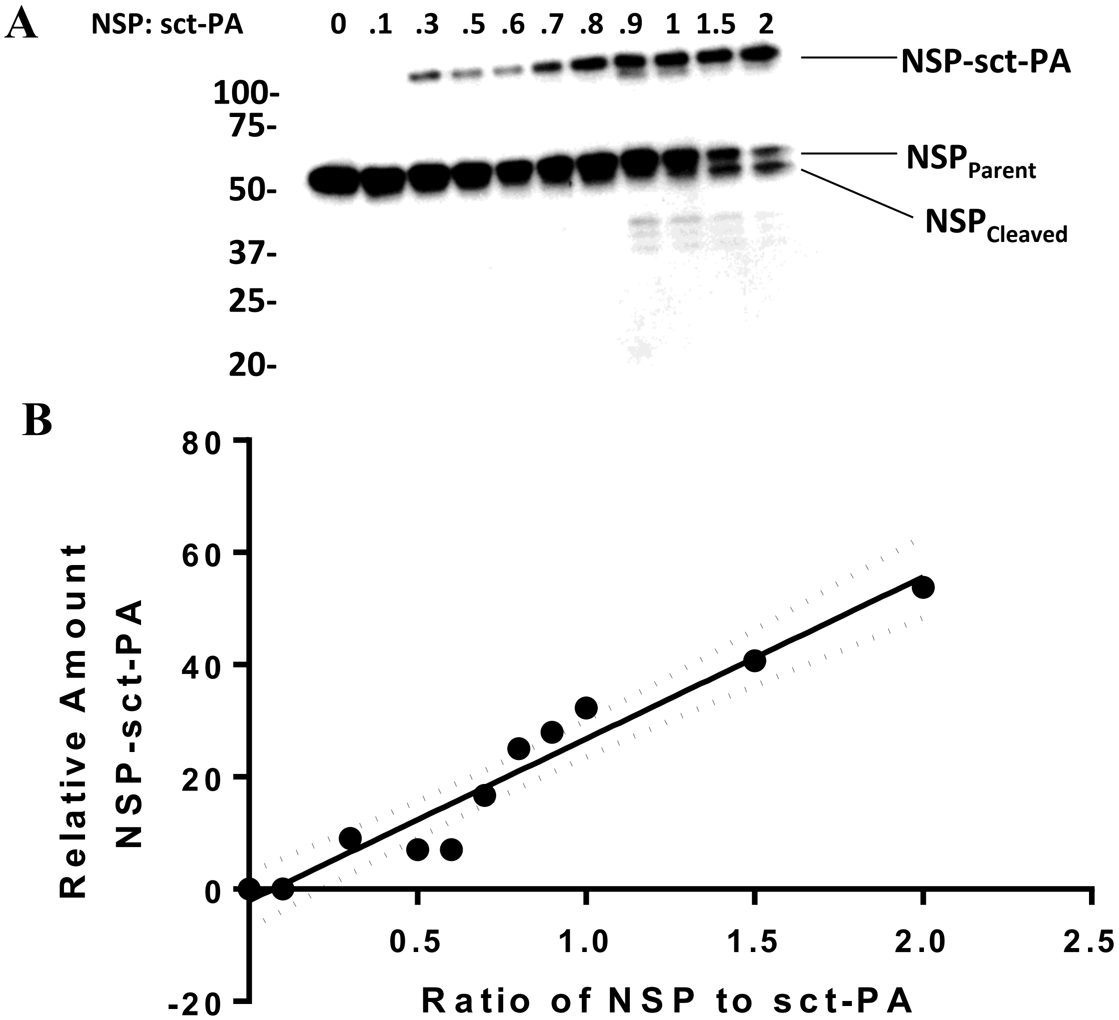

Supplemental Figure 1: *NSP-sct-PA acyl enzyme complex standard curve is linear across the analyzed dose range.* **(A)** Sct-PA was incubated with NSP on ice for 21 hours at pH 7.2 and then samples were subjected to SDS-PAGE under reducing conditions followed by Western blotting for NSP antigen. NSP dose was held constant at 14nM while sct-PA concentration varied from 1.4nM to 28nM. The molar ratio of NSP: sct-PA is indicated in each lane. **(B)** The relative amount of NSP-sct-PA complexes were then compared to the ratio of NSP: sct-PA. NSP-sct-PA complexes, parent band, and cleaved bands were digitally quantified, and the amount of NSP-sct-PA complex relative to total NSP was determined. In this scale, 100 is equivalent to the total amount of immunoreactive NSP. Linear regression analysis using Prism GraphPad software was performed and the calculated R^2 value for linearity was 0.94.
